# New Images for Old Symbols: Meanings That Children Give to a Traditional Game

**DOI:** 10.3389/fpsyg.2021.676590

**Published:** 2021-05-11

**Authors:** Alfonso García-Monge, Henar Rodríguez-Navarro, Daniel Bores-García

**Affiliations:** ^1^Transdisciplinary Education Research Center (CETIE), University of Valladolid, Valladolid, Spain; ^2^Transdisciplinary Education Research Center (CETIE), Department of Pedagogy, University of Valladolid, Valladolid, Spain; ^3^Research Group of Humanities and Qualitative Research in Health Science (Hum&QRinHS), Rey Juan Carlos University, Madrid, Spain

**Keywords:** traditional sporting games, intangible cultural heritage, ethnomotricity, cultural meanings in games, children’s culture, enculturation

## Abstract

Traditional games are considered agents of enculturation. This article explores the procedure to access the cultural meanings transmitted in a traditional game. The goal is to understand what children aged 6–11 make of the game called ‘the chained bear’ and to compare the meanings retrieved with those of different traditional versions of the game. For such a purpose, through an exploratory cross-sectional study, cartoons depicting people playing the game were exhibited and viewers (*n* = 359; age range: 6–11; Mean age = 8.79; *SD* = 1.81) were asked to interpret them as a drama play, as well as contributing a title, a plot and saying how they would name the characters. The results show that, beyond the individual images that each child created in their mind, most of them coincided in stories about harassment and defense and theft and protection. These plots match those of the ludic tradition, showing that the actions evoke different pictures to each individual, but share common cultural meanings in turn. The study shows a procedure to access the meanings that traditional games transmit and confirms that games contain pieces of culture, which makes them agents of enculturation.

## Introduction

As Parlebas (2020) points out: ‘Traditional games are the fruit of a history that has shaped their structures according to the values and collective representations of each region. So, we can expect that universals be in the image of the culture they belong to: Games’ morphology entails cultural meaning’. Many studies have tried to investigate how games collaborate in enculturation processes (e.g., Roberts et al., 1959; Roberts and Sutton-Smith, 1962; Parlebas, 1988). In this article, following Pelegrín (1996) hypothesis of games as traditional gestural drama text, our aim is to understand the interpretation that primary school children make of a traditional game and to look for relationships with the meanings of the game in the ludic tradition. To this purpose, we posed two research questions:

• What do boys and girls interpret when observing the development of a traditional game as if it were a drama performance?
• Do their interpretations have any relation with the plots of the ludic traditions as reflected in the old titles of the games and rhymes that accompanied them?

The initiative, led in the middle of the 20th century by Murdock (1940), to create a database (‘Cross-Cultural Survey Files’) with monographs on different cultures of the planet enabled several comparative studies about different cultural topics. As far as games are concerned, he promoted studies seeking relations between the types of games developed in each cultural context and the culture of reference (e.g., Roberts et al., 1959; Roberts and Sutton-Smith, 1962; Chick, 1998; Peregrine, 2008). Later studies would show that those classifications on which the above research was based could even be more complex (e.g., Parlebas, 1988) and that the complexity of the cultural plots showed limitations in this type of correlations (Mkondiwa, 2020). In any case, as to the old suggestion that games could be an agent of enculturation, formulated by Malinowski in his first ethnography of 1922 (Malinowski, 2014), all these studies advanced an idea that Parlebas(1988, p. 114) summarizes well by saying that ‘when playing, children learn their social universe, unknowingly witnessing the culture to which they belong.’

Although the socialization mechanisms have been well-defined in a number of educational psychology studies (e.g., Elkonin, 2005; Bruner et al., 2017), the mechanisms to understand certain cultural values and meanings through play still remain unclear (Mkondiwa, 2020). To access said cultural meanings, linguist and semiologist Ana Pelegrín suggests that games may be analyzed as drama plays, since they may transmit certain cultural messages. This hypothesis may be supported in cultural psychology works such as Elkonin (1985) when highlighting that, in children’s evolution, access to rules is gained through plot-based dramatized play (inspired by Vygostky, Elkonin, 2005, would delve into the issue of symbolism and the relationships among the object, the word and the action in play). Moreover, Pelegrín’s proposal would inspire in symbolic anthropology (Turner, 1974) when highlighting that the ritualized gesture remains in the repertoire of traditional gestures, as successive players repeat the non-verbal code types transmitted with as many transformations as the group may recreate and with the inherited symbolic implications. But what are those inherited ‘symbolic implications’ suggested by Pelegrín or the ‘cultural meanings’ mentioned by Parlebas? Can we access them?

**FIGURE 1:**
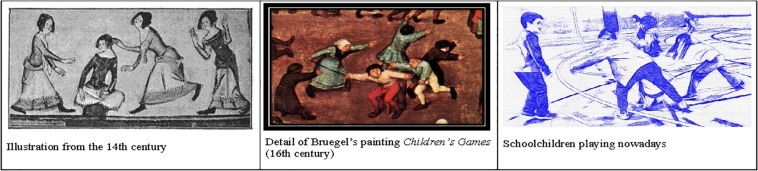
Different representations of the ‘chained bear.’ The gestures have remained the same for centuries, could some parts of the unrevealed symbolic plot be maintained as well?

Much is the literature in which play is picked as a metaphor to describe different life and social situations (e.g., Khayyam, 2003; Tan, 2006; Cortázar, 2008; Gracián, 2014). Different aspects of life are frequently compared with play (Gozzi, 1990). In turn, games and sports have been analyzed as metaphors of different cultural aspects (e.g., Ching, 1993; Jansen and Sabo, 1994; Segrave, 2000; Geertz, 2009). Crawford(2003, p. 29) and Parlebas (2008), each of them from a different viewpoint, seem to agree that play, in some sense, represents something from the non-play universe and therefore is a metaphor. As pointed out by Murray(1997, 136) ‘games can also be read as texts that offer interpretations of experience.’ This is the path pursued by Pelegrín and which we are using in our study: considering the ‘ludic scene’ as a non-verbal representation of a storyline to be interpreted by spectators upon request so that, their interpretations may later be compared with the titles and children’s songs of ludic tradition. For such a purpose, we are choosing a game that has left a historical trace in Europe (as area of cultural diffusion, e.g., Gillmeister, 2009; Lidström and Bjärsholm, 2019; O’Brian, 2019) since the 1st century at least. We are talking about a game that has been called *navero*, ‘chained bear’ (Pelegrín, 1996) or ‘the bear and its guardian’ (Parlebas, 2008), similar to ‘frog in the meadow’ or ‘frog in the middle.’ Pelegrín (1998) has found documentary evidence of the game in different paintings and literary works since the 16th century. As for Parlebas (2008), he has tracked its presence in frescos of Pompeii (dated back to the 1st century), a sarcophagus in the Vatican (3rd century), in Bruegel’s artwork (16th century) and in a tapestry in Paris (18th century) (Figure 1).

A round of this game would consist of one player crouching in the middle, by whom another player stands touching the crouching one’s head. The other players stand out of reach of these two. Those out-of-reach players attempt to touch the crouching one while avoiding being touched by the standing person to the side. Where any of these players gets touched by the standing one, they will have to assume the crouching player’s role. In some traditional versions of the game the link between the crouching player and his or her defender is established with a rope that marks the defender’s radius of action. In other versions, it is a circle marked on the ground that demarcates the defender’s movement.

## Materials and Methods

### Design

Given that this is a first approach to this phenomenon, an exploratory study (Singh, 2007) has been chosen and, in order to know its manifestation at different ages, a cross-sectional design has been developed (Cohen et al., 2012).

### Participants

The sample was selected by non-probabilistic convenience sampling. A total of 359 schoolchildren from three Spanish cities took part in this study (18 groups of children from 6 grades of primary education, Table 1).

**TABLE 1:** Sample data.

Grade	*n*	Mean age (*SD*)	Boys	Girls
1° grade	58	6.31 (*SD* = 0.63)	32 (55.17%)	26 (44.83%)
2° grade	56	7.34 (*SD* = 0.59)	31 (55.36%)	25 (44.64%)
3° grade	62	8.41 (*SD* = 0.58)	30 (48.39%)	32 (51.61%)
4° grade	64	9.21 (*SD* = 0.64)	33 (51.56%)	31 (48.44%)
5° grade	60	10.32 (*SD* = 0.54)	26 (43.33%)	34 (56.67%)
6° grade	59	11.15 (*SD* = 0.66)	30 (50.85%)	29 (49.15%)
Total	359	8.79 (*SD* = 1.81)	182 (50.7%)	177 (49.3%)

The cities in which the information was collected are cities of different sizes (one of 160,000 inhabitants, another of 500,000 inhabitants and another of 6 million inhabitants). In the three schools, located in middle-class neighborhoods of the respective cities, the students belong to varied socio-cultural contexts (in all three, there is a mixture of families with higher, middle and lower educational qualifications). Two of the schools in the sample were chosen because two of the researchers have been working there on a weekly basis for years. The school in the big city was chosen because one of the researchers worked there.

None of these children knew the game in advance.

Informed consent was duly obtained from their schools and families. The study was approved by the Ethics Committee of the University of Valladolid (code: PI 19-1920NOHCUV) in accordance with the Declaration of Helsinki.

### Instruments

Based on previous studies (Segrave, 2000; Elkonin, 2005), a video of some characters playing the game was elaborated. This game was chosen because of the possibilities of tracing it throughout history. This facilitates the comparison of the students’ opinions with tradition. After viewing such a video, participants were asked to answer some questions about the video on a worksheet.

• Three phases were followed in the elaboration of the video used.

First: filming. The characters filmed were university students. With the aim of reducing any potential bias, all characters were dressed in the same colors (sports clothes without logos or prints) and showed neutral facial expressions. The image background was a light color wall on which the actors’ figures stood out. The video showed a group made up of 5 individuals. One of the individuals was in a crouch. Another person, standing beside the crouching one, was touching the latter’s head. Finally, the three remaining individuals were standing around these two. These three individuals, forming a semicircle, would attempt to get closer to the crouching character and try to touch them, while avoiding being touched by the one standing by.

Second: the video was transformed into cartoons through an image processing application (*Clips* app).

Third: The video was previously tested with two focus groups of 20 children aged 7–9 (*M* = 8.5, *SD* = 0.93; *M* = 8.4; *SD* = 0.95). Participants had to watch the video twice and, after that, give a title to the scene, describe the storyline and name the characters. In this previous test, actions, facial gestures or clothing did not reveal significant bias. Also, the video was provided with the proper length for viewers to capture the necessary information without becoming tired or distracted.

**FIGURE 2:**
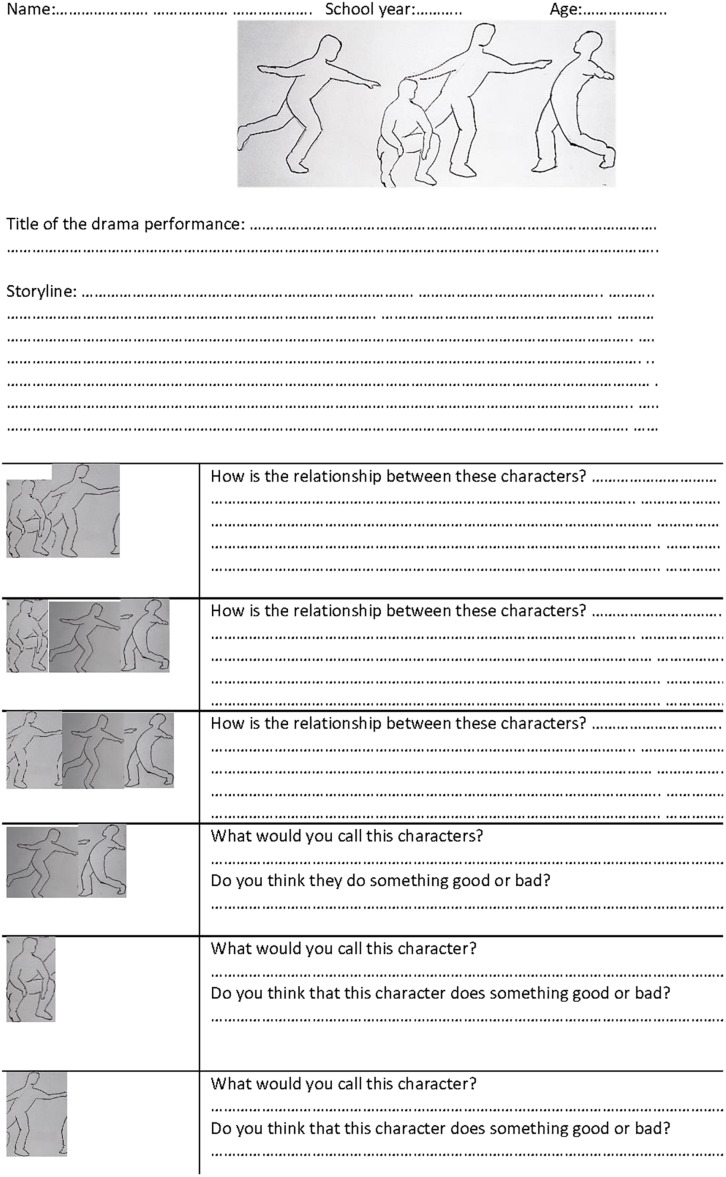
English version of the worksheet used for data collection.

• The data collection worksheet was elaborated from the analysis categories suggested by Pelegrín (1998), including a schematic drawing of the players (without facial expressions or specific clothes) and some sections for the children to add a title to the video, write a brief narration of what was happening and a description of the relationships between the characters, name the different characters and rate their actions as “good” or “bad.” After its initial design, this instrument was validated by 5 experts (Fleiss’ κ = 0.95). Following the procedure proposed by Escobar-Pérez and Cuervo-Martínez (2008), each section of the worksheet was evaluated (“does not meet the criterion,” “low level,” “moderate level,” and “high level”) in three aspects (clarity, coherence, and relevance). Following that, the worksheet was tested on a focus group of 15 children aged 7 and 8 (*M* = 7.81; *SD* = 0.53) and the necessary amendments were made. The new changes were again validated by a group of 5 experts (Fleiss’ κ = 1). The drawing of the first version showed people with neutral facial expression, even so, some children attributed emotional states to them; to avoid this, the drawings were replaced by human silhouettes without facial expression. Furthermore, in the preliminary test it was found that the space for the answers could be small given the size of the children’s font (Figure 2).

### Procedure

The physical education teachers, together with the researchers, designed a teaching unit about traditional games. The initial task of the first lesson consisted in collecting the students’ opinions about the meanings they gave to the game of “chained bear” after watching a video in which some people played it. After this task, the students play the game themselves and, in teams, modified some rules to make it more inclusive and safer.

Data collection was performed in the classrooms of the different schoolchildren involved, as follows:

• A researcher explained to the group that they were going to watch a story on video. After that, they would have to find a title for the story and tell what they thought was happening there, while insisting that there was not any right solution and that it was just a way to see what each of them had in their mind.
• The video was played for the first time.
• The children were reminded that they should find a title which reflected what was happening in the story and that they could think of the role each actor was playing.
• Next, the children watched the video for a second time.
• Followingly, they were given out sheets and explained their different sections.

The researchers and the teachers were watchful of any children with difficulties to understand the task or even to write (specially the children aged 6–7). Many participants left questions unanswered (each child left different questions unanswered). After confirming that they had properly understood the task, some pupils simply did not know what to answer and were allowed to write a response to any questions that they wished in order to avoid forced answers.

During their explanations, the researchers avoided using words which could contain connotations about the characters or the situation (e.g., ‘attacker,’ ‘defender,’ ‘protect,’ etc.), thus restricting themselves to define them just by their location in the space.

All groups completed the task in less than 15 min.

### Analysis

The results were sorted out in a table by ages. Atlas.ti 8. software was used to facilitate the categorization and analysis of the content. The analysis of results followed Pelegrín (1996) proposal to study the semantic and pragmatic planes of the subjects’ interpretations in a contrast between the level that Turner (2005) calls exegetic (the subjects’ own perspective) and the positional one (the researchers’ interpretation to unveil the meaning). In this case, a positional analysis would lie in the contrast between the subjects’ interpretations and the traditional ludic plots or maps as collected in Pelegrín’s works (1996, 1998). Independent significance analyses were performed by each of the researchers and consistency amongst researchers was tested (Concordance = 98.2% [no. agreements − no. disagreements) × 100/Total no. of concepts]). According to Van Dijk (1990), these plots, maps or frameworks denote a certain conceptual structure in semantic memory and represent a part of our knowledge about the world, relating convention to experience. Following this author, discourse shows not only cognition, but also the speaker’s cultural contexts of reference.

## Results

With the aim of enhancing the exhibition and discussion of the results, we shall refer to the person in a crouch as the ‘crouching person or player,’ the standing person next to the latter as ‘the defender’ and the rest of the participants as ‘the attackers’.

Table 2 shows the names given by the study participants to the different characters, sorted out by ages and the positive or negative rates awarded.

**TABLE 2:** Names given by the study participants to the different characters, sorted out by ages and the positive or negative rates awarded.

Characters’ names given by the 6-year-olds											
Attackers				Defender				Crouching player			
Positive		Negative		Positive		Negative		Positive		Negative	
Name	No.	Name	No.	Name	No.	Name	No.	Name	No.	Name	No.
‘Saviors’	5	Spies’	3	‘Protector’	5	‘The evil one’	4	‘Boy’	4		
‘The good ones’	3	‘Superpunches’		‘Carer’	4	‘Wolf’		‘Girl’	2		
‘Touchers’	3	‘Punches’		‘Squire’	3	*‘Push-asider’*		‘Baby’	3		
‘Blowing colors’		‘Iron fist’		‘Defender’	2	‘Guard’ 3	3	‘Princess’			
‘Children playing’	2	‘Fire fists’		‘Guardian’	6	‘Witch’ 3	3	‘Sleepyhead’			
‘Twisters’		‘Thieves’	4	‘Police’	6	‘Dragon’		‘The sick one’			
		‘Attackers’	3	‘Father’	3			‘Mate’	3		
		‘The evil ones’	5	‘Mother’	2			‘The drowned one’			
		‘Mosquitoes’	2	‘Guard’				‘Snowman’			
		‘Bees’		‘Wind’				‘Turnip’			
		‘Wolves’	4	‘Spinning girl’				‘Flower’			
		‘Mice’		‘Protecting kid’				‘Bloke’			
		‘Pirates		‘Cat’				‘Lightbulb’			
								‘The good one’			
								‘Dog’	4		
								‘Puppy’			
								‘Frog’	2		
								‘Owl’			
								‘Food’			
								‘Cheese’			
								‘Treasure’	2		
								‘Lion’			
								‘Bear’			
15 (25.86%)		28 (48.27%)		36 (62.06%)		13 (22.41%)		36 (62.06%)			

**Characters’ names given by the 7-year-olds**																					
**Attackers**				**Defender**				**Crouching player**			
**Positive**		**Negative**		**Positive**		**Negative**		**Positive**		**Negative**	
**Name**	**No.**	**Name**	**No.**	**Name**	**No.**	**Name**	**No.**	**Name**	**No.**	**Name**	**No.**

‘Touchers’	2	‘The evil ones’	3	‘Mosquito hunter’		‘Toucher’		‘The good one’	2		
‘Adults’		‘Enemies’	3	‘The good one’	2	‘Guard’	2	‘The little one’			
‘Parents’		‘Dog attackers’	3	‘Guardian’	6	‘Doctor Flux’		‘Mate’	2		
‘Children’	3	‘Thieves’	5	‘Defender’	7	‘Warden’		‘Rock’	2		
‘Happy girls’		‘Attackers’	6	‘Shield’		‘Monster’		‘The sleepy one’			
‘Supergirls’		‘The evil ones’	3	‘Protector’	4			‘Kid’	6		
‘Girls playing with a dog’		‘Fools’		‘Toucher’				‘Girl’	3		
‘Sailors’		‘Murderers’	2	*‘Touchator’*				‘Baby’	3		
‘Fishers’		‘Fight’		‘Superman’				‘Food’			
‘Incredibles’		‘Wolves’	2	‘Civil guard’				‘Frog’	2		
‘Rescuing cops’		‘Skeletons’		‘Kid’				‘Dog’	8		
‘Rescuers’		‘Monsters’		‘Dog owner’				‘Fish’			
		‘Pirates’		‘Animal-protecting guardian’				‘Machine’	2		
		‘Mosquitoes’	3	‘Dog guardian’				‘Treasure’	4		
				‘Dog-protecting kid’				‘Prisoner’	3		
				‘Spider’	2			‘The sick one’	2		
15 (26.78%)		35 (62.5%)		32 (57.14%)		6 (10.71%)		43 (76.78%)			

**Characters’ names given by the 8-year-olds**											
**Attackers**				**Defender**				**Crouching player**			
**Positive**		**Negative**		**Positive**		**Negative**		**Positive**		**Negative**	
**Name**	**No.**	**Name**	**No.**	**Name**	**No.**	**Name**	**No.**	**Name**	**No.**	**Name**	**No.**

‘Touchers’	2	‘Infectors’		‘World savior’		‘The evil one’	2	‘King’	2	‘The jailed one’	
‘Children’	2	‘The evil ones’	4	‘Soldier’		‘Terrorist’		‘Queen’			
‘Treasurers’		‘Aggressors’		‘Catcher’		‘Kid’		‘Princess’			
‘The good ones’		‘Disturbers’		‘The good one’	2	‘Witch’		‘Beautiful house’			
‘Prisoner’s friends’		‘Attackers’	12	‘Kid’	2			‘Kid’	10		
‘Children’	4	‘Punchers’		‘Protector’	8			‘Baby’	2		
‘Girls’		‘Teasers’		‘Batman’				‘Mate’			
‘Parents’		‘Mosquitoes’	2	‘Defender’	9			‘The good one’			
		‘The *pain-in-the-necks’*		‘Guard’	4			‘Trapped kid’			
		‘Thieves’	6	‘Referee’				‘Protected kid’ ‘Scared kid’			
		‘Muggers’	4	‘Guardian’	8			‘Prisoner’			
		‘Wolves’		‘Doctor’				‘Treasure chest with golden coins’			
**Attackers**				**Defender**				**Crouching player**			
**Positive**		**Negative**		**Positive**		**Negative**		**Positive**		**Negative**	
**Name**	**No.**	**Name**	**No.**	**Name**	**No.**	**Name**	**No.**	**Name**	**No.**	**Name**	**No.**
		‘Lions’	3	‘Father’				‘Chest’			
		‘The Creeper’		‘Mother’				‘Treasure’	4		
		‘Family’		‘Dog owner’				‘Jewel’	2		
		‘Thieves’ family’		‘Galactic guardian’				‘Energy stone’			
		‘Attackers from the future’						‘The protected one’			
		‘Tornado’						‘The annoying one’			
		‘Kooks’						‘Defenseless man’			
		*‘Botherers’*						‘Captain America’			
		‘Galactic fighters’						‘The weak one’			
								‘Precious stone’			
								‘Table with food’			
								‘The secret’			
								‘Frog’	2		
								‘Dog’	5		
13 (20.96%)		45 (72.58%)		43 (69.35%)		5 (8.06%)		47 (75.8%)		1 (1.61%)	

**Characters’ names given by the 9-year-olds**											
**Attackers**				**Defender**				**Crouching player**			
**Positive**		**Negative**		**Positive**		**Negative**		**Positive**		**Negative**	
**Name**	**No.**	**Name**	**No.**	**Name**	**No.**	**Name**	**No.**	**Name**	**No.**	**Name**	**No.**

‘Friends’		‘Thieves’	6	‘Cop’	2	‘Bad hoarding child’		‘Roman king’		‘Burglar’	
‘Two generous children’		‘Children’	2	‘Protecting man’		‘Devil’		‘King’	2	‘Local traitor’	
‘Superheroes’		‘The evil ones’		‘Protector’	6			‘King’s defender’		‘Magic stone with powers to transform you’	
‘Family’		‘Deer hunters’		‘Mother’				‘Friend’	2		
‘Girls’		‘Kidnappers’		‘Defender’	12			*‘Silly-person’*			
‘Runners’		‘The *pain-in-the-necks’*		‘Lifesaver’				‘Prisoner’			
		‘Attackers’	11	‘Doctor’				‘Prisoner’	5		
		‘Bullies’		‘Carer’	2			‘Kid’	6		
		‘Harassers’		‘Guardian’	7			‘Sad kid’			
		‘Pesterers’		‘Guard’	4			‘Kid whose’ watch others’ want to steal’			
		‘Mosquitoes’		‘Magician’				‘Little thing’			
		‘Crazy catchers’		‘Kid’				‘The sick one’			
		‘Gluttons’		‘Protecting kid’				‘The weak one’			
				‘Child that has gone kind’				‘Scared deer’			
				‘Helping kid’				‘Bull’			
				‘Catcher’	3			‘Frog’			
				‘Cook’				‘Dog’			
				‘Seller’				‘Father’s money’			
				‘Gardener’				‘Last *rukifruc* (worth a fortune)’			
								‘Golden statuette with powers’			
								‘Golden stone’			
								‘Wedding ring’			
								‘Special thing’			
								‘Star’			
								‘Lucky statue’			
								‘Valuable stone’			
								‘Diamond’			
								‘Stone’	2		
								‘Food pot’			
								‘Chocolate’			
								‘Food’			
								‘Cabbage’			
6 (9.37%)		29 (45.31%)		48 (75%)		2 (3.12%)		44 (68.75%)		3 (4.68%)	

**Characters’ names given by the 10-year-olds**											
**Attackers**				**Defender**				**Crouching player**			
**Positive**		**Negative**		**Positive**		**Negative**		**Positive**		**Negative**	
**Name**	**No.**	**Name**	**No.**	**Name**	**No.**	**Name**	**No.**	**Name**	**No.**	**Name**	**No.**

‘Children’		‘The evil ones’	2	‘Friend’		‘The evil one’		‘Kid’		‘Hostage’	
‘Cops’		‘Evildoers’		‘Defender’	14	‘Buddy’		‘Friend’	2	‘Thief’	
		‘Wrongdoers’		‘Carer’	4	‘The touchy one’		‘Mate’		‘Magnet stone’	
		‘Thieves’	6	‘Protector’	10			*‘Defendman’*			
		‘Attackers’	16	‘Guardian’	5			‘Cutie’			
		‘Buddies’		‘Catcher’	2			‘Hostage’	4		
		‘Children’	2	‘Children’	4			‘Prisoner’	5		
		‘Virus carriers’		‘Doctor’				‘Prisoner girl’			
		‘Viruses’		‘Beekeeper’				‘Boulder’			
		‘Harassers’						‘Flag’			
		‘Bullies’						‘Jewel’			
		‘Twister’						‘Power stone’			
		‘Wisecracks’						‘Base’	2		
		‘Hagglers’						‘Sheep’			
		‘Idiots’						‘Dog’			
		‘Bear’						‘Frog’	3		
								‘Penguin’			
								‘Pineapple’			
								‘Honey’			
2 (3.33%)		38 (63.33%)		42 (70%)		3 (5%)		30 (50%)		3 (5%)	

**Characters’ names given by the 11-year-olds**											
**Attackers**				**Defender**				**Crouching player**			
**Positive**		**Negative**		**Positive**		**Negative**		**Positive**		**Negative**		
**Name**	**No.**	**Name**	**No.**	**Name**	**No.**	**Name**	**No.**	**Name**	**No.**	**Name**	**No.**

‘Mate’		‘Thugs’		‘Mate’	4	‘Abductor’		‘Mate’	2	‘Statue’	2
*‘Touchator’*		‘Thieves’	4	‘Friend’		‘Monster’		‘Friend’		‘Magnet’	
‘Parents’		‘Attackers’	15	‘Doctor’				‘The one everybody tries to kill’			
‘Horses’		‘Aggressors’		‘Bodyguard’	6			‘Protected’	4		
‘Children playing’	9	‘Fidgeters’		‘Defender’	14			‘Kid’	6		
*‘Head-poker’*		‘Guys who are trying to kill’		‘Shepherd’				‘Kidnapped kid’			
		‘Murderers’		‘Guardian’	10			‘Prisoner’	2		
		‘Bullies’		‘The bitter one’				‘Victim’			
		‘Wolves’		‘Mr *do-not-let-others-ride-the-mare’*				‘Treasure’	4		
		‘Children’		‘Catcher’	6			‘Sheep’			
		‘Fans’						‘Donkey’			
		‘Horses’						‘Mare’			
								‘Frog’	2		
								‘Famous frog’			
								‘Diamond frog’			
								‘Flag’			
								‘Valuable rock’			
								‘Golden stone’			
								‘Meteorite’			
								‘Statue’			
								‘Doorbell’			
14 (23.72%)		29 (49.15%)		45 (76.27%)		2 (3.38%)		35 (59.32%)		3 (5.08%)	

As can be seen, there is a wide range of names, as well as negative and positive ratings for the characters. In all ages, the widest range of names can be found in the crouching player’s column, despite the fact that they do not actually move. The largest number of ratings can be found between 8 and 9 years old.

Images evoke different associations in each observer, some of which reflect the current cultural environment of these children (‘pirates,’ ‘batman,’ ‘galactic fighters,’ etc.).

Among the children who have provided an answer, the crouching player is hardly ever associated with negative values. The attackers tend to be perceived as negative and the defender is mostly regarded as positive.

The crouching player: this character has been given names that relate to their size and position (‘dog,’ ‘frog,’ ‘stone,’ ‘kid,’ ‘baby,’ ‘snowman,’ etc.), on their defenselessness and trapped nature (‘baby,’ ‘the sick one,’ ‘the weak one,’ ‘prisoner,’ ‘defenseless man,’ ‘scared kid,’ etc.), on a possible connection with power (‘king,’ ‘queen,’ ‘princess,’ ‘bear,’ ‘bull,’ etc.), or with worth (‘gold,’ ‘jewel,’ ‘diamond,’ ‘treasure,’ etc.) and on something to be stolen (‘food,’ ‘cheese,’ ‘turnip,’ ‘cabbage,’ etc.). Some children have explained the fact that attackers feel attracted to touch the crouching player by defining the latter as a magnet, or else a powered or a magical stone which transforms everybody who touches it.

Attackers: most meanings awarded to these characters are associated with harassment and aggression (‘mosquitoes,’ ‘fire fists,’ ‘bees,’ ‘wolves,’ ‘bullies,’ ‘stalkers,’ ‘fans,’ etc.), theft (‘thieves,’ ‘mice,’ ‘pirates,’ ‘kidnappers,’ etc.) or, on the contrary, rescue (‘family,’ ‘friends,’ ‘rescuers,’ ‘saviors,’ ‘cops,’ etc.).

Defenders: this character is usually regarded as a protector or carer (‘doctor,’ ‘father,’ ‘mother,’ ‘shepherd,’ ‘protector,’ ‘defender,’ ‘protecting kid,’ ‘carer,’ etc.), or even as a keeper (‘cop,’ ‘beekeeper,’ ‘guardian,’ ‘guard,’ ‘gardener,’ ‘seller’), although this last connotation can also be regarded negatively (‘warden,’ ‘abductor,’ ‘bad hoarding child,’ ‘witch,’ ‘terrorist,’ etc.).

These names define the main plot lines and themes: protection and defense against harassment, defense of property, person or animal, and rescuing someone who has been captured. However, there are some troubling themes imagined by participants, in which the attackers see themselves attracted to their ruin by the central characters. As shall be analyzed below, these plots match different versions of this game in the ludic European tradition.

## Discussion

Games are considered enculturation agents. This study explores how to potentially transmit culture through play. Following Pelegrín (1996) hypothesis of games as traditional gestural drama text, our aim is to understand the interpretation that primary school children make of a traditional game and to look for relationships with the meanings of the game in the ludic tradition.

In this section, we shall compare our results with the document sources cited, especially Pelegrín (1996, 1998), with the aim of analyzing whether this game can show some permanent meanings.

The crouching character is a major focus of attention, as happens in the tradition (Pelegrín, 1996). This character is the central part of the scene, where all players’ actions converge. Their importance is evidenced by the fact that this character appears in the title of many of the traditional versions of the game (‘chained bear,’ ‘pot,’ ‘penguin,’ *cucula*, ‘frog in the middle’). The connotations awarded by the subjects are similar to the traditional ones. The character’s defenselessness (*poiré*, ‘kid,’ and ‘attacked’), worth (‘honey pot,’ ‘fruit,’ and ‘vegetable’) or power (‘chained bear,’ ‘tied bull,’ *cucula*, and ‘chained devil’), as well as some other specific names of the ludic tradition match those awarded by the subjects (‘kid,’ ‘frog,’ ‘penguin,’ ‘honey,’ ‘bull,’ ‘turnip,’ and ‘cabbage’).

The meanings given to the attackers are also related to those of the ludic tradition (‘thieves,’ ‘attackers,’ and ‘aggressors’), except the connotation provided by some of the subjects of this study as rescuers or helpers of the crouching player. It could be inferred that, even though this scene can be construed like this when observed as a representation, when analyzing it as a game, it can be clearly appreciated that the attackers’ aim is not to help, despite the fact that their actions result in an indirect way of helping (they put themselves at risk and, if they are captured, replace the crouching character in their position). This new view introduced by the participants of this study may be revealing a cultural change, according to the process described by Elschenbroich (1979) or Elias and Dunning (1992) in other forms of physical practices. Strong and aggressive contact was most frequent in traditional games (V. A., 2001) and, in fact, games played in a circle around a central character were likely to end up in harassment and mockery (‘botfly,’ ‘guess who poked you,’ ‘amusement,’ *poiré*; V. A. 2001, Pelegrín, 1998).

Furthermore, the defender is usually conceived by the ludic tradition as a protector and guardian in the shape of the owner of the ‘bear,’ ‘the owner of a monkey,’ ‘truck gardener,’ ‘gardener,’ ‘seller,’ or ‘guardian of the pot’ (Pelegrín, 1996, 1998). However, the tradition does not show any negative connotations about this character (at least, directly).

The plot lines and themes, to wit, protection and defense against harassment, defense of property, person or animal, and rescuing someone who has been captured, match those of the ludic tradition. That does not happen with the idea of helping the captive. Bonhome (1989, op. cit in Pelegrín, 1996) deduces that the game layout may evoke certain rites of Ancient Greece and Rome, where a messenger from hell would transfer their powers to whomever touched them, something represented by the participants when they called the crouching person a ‘statue,’ ‘magnet,’ or ‘stone which transforms you,’ guarded by a ‘witch,’ ‘monster,’ or ‘devil’. As indicated by Pelegrín(1996, p. 96), play is a reflection of ‘inexhaustible ancient images full of symbolism; even when their meaning is uncertain, symbols open a vast network of possibilities for imagination.’

The results show that the actions evoke a different image in each person. Nonetheless, the plot meanings seem to be reduced to just a few ones, common amongst the participants and also to those of the tradition. Beyond any personal connotations, common cultural patterns and shared meanings can be perceived, which evidences that these traditional meanings exist in our society, since, as suggested by Turner (2005) they are still understandable and recognizable by children nowadays.

This experiment would contribute evidence to Pelegrín’s hypothesis: traditional games evoke cultural images, stories and meanings through spatial and gestural display. That is where one of the values of preserving traditional games arises, since, as stated by Parlebas(2001, p. 223), ‘games are in accordance with the culture which they belong to, especially as regards the features of their internal logic, features that illustrate values and symbols underlying the said culture.’ This is possibly due to the fact that play always revolves around interpersonal relationships and their social-historical background, as suggested by Elkonin (1985) and Vygotsky (2016).

Besides, this study shows a procedure to access some of these meanings evoked in the participants by the ludic actions. To better understand these meanings, the plot lines of the game should be addressed from concept frameworks such as those used by Propp in the study of tales, which has already been used to analyze symbolic games (Navarro, 2005).

This could be suggested as a future line of research in view of the existing relationship between meanings and emotions. A relation has been found between certain types of games and certain emotions (Lavega et al., 2014; Hedegaard, 2016; Alcaraz-Muñoz et al., 2020) and future studies could perfectly address links between the meanings of the games and the emotions awakened in participants (following Turner’s (2005), notion of symbols as stimuli of emotions).

Although the study shows that children observe common cultural patterns and shared meanings with the tradition from observing the game, it remains to be seen whether these meanings are the same when observing a game and when playing the game. Cultural immersion is a process in which both phases (first observation and then participation and transformation) normally occur, but this question opens up new directions for inquiry. Nowadays, we are studying how perceptions toward a game becomes different once it has been played, in other words, how participants call the game itself and the different roles played in it.

## Data Availability Statement

The original contributions generated for this study are included in the article/supplementary material, further inquiries can be directed to the corresponding author.

## Ethics Statement

The studies involving human participants were reviewed and approved by Valladolid University Ethics Committee. Written informed consent to participate in this study was provided by the participants’ legal guardian/next of kin.

## Author Contributions

AG-M: theoretical background and design. AG-M, HR-N, and DB-G: data collection, data analysis, and writing of the final manuscript. All authors contributed to the article and approved the submitted version.

## Conflict of Interest

The authors declare that the research was conducted in the absence of any commercial or financial relationships that could be construed as a potential conflict of interest.
